# Enhancing comprehensive genome profiling of liver tumors using endoscopic ultrasound-guided fine-needle biopsy

**DOI:** 10.1055/a-2712-9822

**Published:** 2025-10-23

**Authors:** Fumitaka Niiya, Akihiro Nakamura, Yasuo Ueda, Takafumi Ogawa, Naoki Tamai, Masataka Yamawaki, Jun Noda, Tetsushi Azami, Yuichi Takano, Masatsugu Nagahama

**Affiliations:** 1 26858 Division of Gastroenterology, Department of Internal Medicine, Showa Medical University Fujigaoka Hospital, Yokohama, Japan; 2 26858 Division of Pathology, Showa Medical University Fujigaoka Hospital, Yokohama, Japan

**Keywords:** Endoscopic ultrasonography, Tissue diagnosis, Fine-needle aspiration/biopsy, Quality and logistical aspects, Performance and complications

## Abstract

**Background and study aims:**

Comprehensive genome profiling (CGP) is gaining importance in management of biliary and pancreatic cancers; therefore, obtaining ideal tissue samples is critical. Although percutaneous biopsy has been the first-line method of sampling liver tumors, recent advances in endoscopic ultrasound (EUS)-guided tissue acquisition (EUS-TA), particularly introduction of fine-needle biopsy (FNB) needles capable of obtaining larger tissue samples, suggest that EUS-TA may be an alternative for obtaining ideal specimens for CGP. However, few studies have evaluated the utility of EUS-TA with CGP for liver tumors. This study aimed to assess the role of EUS-guided FNB (EUS-FNB) in collection of ideal liver tumor samples for CGP and analyses.

**Patients and methods:**

This retrospective study included 36 patients with liver tumors who underwent EUS-FNB. Histological diagnostic accuracy, rate of obtaining ideal samples for CGP, and impact of procedural factors, such as needle gauge, tumor size, and number of punctures, were analyzed.

**Results:**

EUS-TA achieved a histological diagnostic accuracy rate of 94.4%, and the rate of obtaining ideal samples for CGP was 63.9%. Subgroup analysis showed that 22G (70.4%) and 19G (100%) needles were significantly more effective than 25G needles (16.7%) for obtaining ideal samples for CGP ( *P* =0.025 and *P* =0.048, respectively). No adverse events were observed during or after sampling.

**Conclusions:**

EUS-FNB is highly effective for obtaining ideal samples for CGP and achieving an accurate histological diagnosis. The 22G and 19G needles were significantly superior to 25G needles, thus emphasizing their importance in precision medicine.

## Introduction

Comprehensive genome profiling (CGP) using next-generation sequencing has been applied to numerous solid tumors because genotype-specific therapies can enhance prognoses 1 2 . CGP was first covered by insurance in Japan in 2019; since that time, its adoption has expanded significantly and companion diagnostic devices, such as the Onco-Guide NCC Oncopanel System (Sysmex Corporation, Hyogo, Japan) and FoundationOne CDx (F1CDx; Foundation Medicine, Cambridge, MA, USA), have been utilized for unresectable solid cancers that are refractory to standard therapies. However, because CGP requires a substantial amount of tissue, it is important to evaluate and optimize tissue sampling methods.

Endoscopic ultrasound (EUS)-guided tissue acquisition (EUS-TA) has been used extensively to diagnose gastrointestinal lesions and those in adjacent regions 3 . Several studies have demonstrated CGP success rates of 39.2% to 97.0% using samples collected via EUS-TA 4 5 6 7 8 9 10 . Therefore, EUS-TA may be a valuable method of sampling tissue for CGP.

Unlike pancreatic tumors, liver tumors such as metastatic lesions, hepatocellular carcinoma, and cholangiocarcinoma frequently exhibit central necrosis and heterogeneous cellularity. These histological characteristics may affect the adequacy of samples for genomic profiling, underscoring the need for liver-specific evaluations. Percutaneous biopsy is the primary method of obtaining liver tumor samples for CGP, mainly because it allows collection of larger tissue samples. However, the 2021 European Society of Gastrointestinal Endoscopy guidelines only weakly recommend EUS-guided biopsy for liver tumors and suggest its use solely for exceptional circumstances such as anatomical challenges or percutaneous biopsy failure because of the established efficacy of percutaneous liver biopsy for obtaining tissue samples from liver tumors 11 . Although percutaneous biopsy allows collection of larger tissue samples, EUS-TA offers several advantages. For example, although EUS-TA needles are smaller (19G-25G) than those used for percutaneous liver biopsy (16G-18G), EUS provides high spatial resolution that enables avoidance of small vessels and is not affected by subcutaneous fat or intestinal tract. A study that compared the diagnostic effectiveness of percutaneous liver biopsy and EUS-TA for liver tumors found that both methods had comparable sensitivity, specificity, and diagnostic accuracy. However, significantly fewer complications were observed with EUS-TA 12 . Recently, EUS-guided fine-needle biopsy (FNB) using core biopsy needles has been introduced to obtain core tissues that are more suitable for histological evaluations 13 . In addition, FNB contributes to successful next-generation sequencing analyses 4 . Therefore, EUS-TA using FNB needles may be a valuable method of obtaining liver tumor tissue samples for CGP. Despite these potential advantages, few studies have specifically examined the efficacy of EUS-TA using FNB needles to obtain liver tumor samples for CGP. Therefore, we evaluated the utility of EUS-TA with FNB needles for liver tumors.

## Patients and methods

Ethical approval for this study was obtained from the Institutional Review Board of the hospital (2024–224-B). This research was conducted in accordance with the principles of the Declaration of Helsinki.

We conducted a retrospective analysis of patients who underwent EUS-TA using FNB needles for liver tumors at Showa Medical University Fujigaoka Hospital between December 2021 and August 2024. Exclusion criteria were: 1) EUS-guided liver biopsy for diffuse liver disease; 2) benign tumors; and 3) insufficient clinical data for outcome adjudication. Consequently, the final analysis was restricted to patients who were ultimately diagnosed with malignant tumors.

### EUS-TA procedure

All EUS-TA procedures were performed under sedation with midazolam (1–5 mg) and pethidine hydrochloride (35 mg) by four endoscopists with more than 4 years of experience. An oblique forward-viewing electronic linear scanning video echoendoscope (GF-UCT260; Olympus Medical Systems Corp., Tokyo, Japan) and observation devices (UE-ME1 and UE-ME2; Olympus) were used. For EUS-TA, a 19G FNB needle (Acquire; Boston Scientific, Tokyo, Japan), 25G FNB needle (Acquire; Boston Scientific), or 22G needle (SonoTip TopGain; Medico’s Hirata, Tokyo, Japan) was used at the discretion of the endoscopist. The puncture procedure involved 10 to 20 strokes performed under negative pressure of 20 mL or using the slow-pull method, depending on endoscopist preference. The endoscopist determined the number of punctures. Rapid onsite evaluation was not performed during this study. The collected specimens were immediately immersed in bottles containing a 10% formalin solution. For cases involving antithrombotic medication use, EUS-TA was performed in accordance with guidelines of the Japanese Gastroenterological Endoscopy Society 14 .

### Pathological assessment

**Fig. 1:**
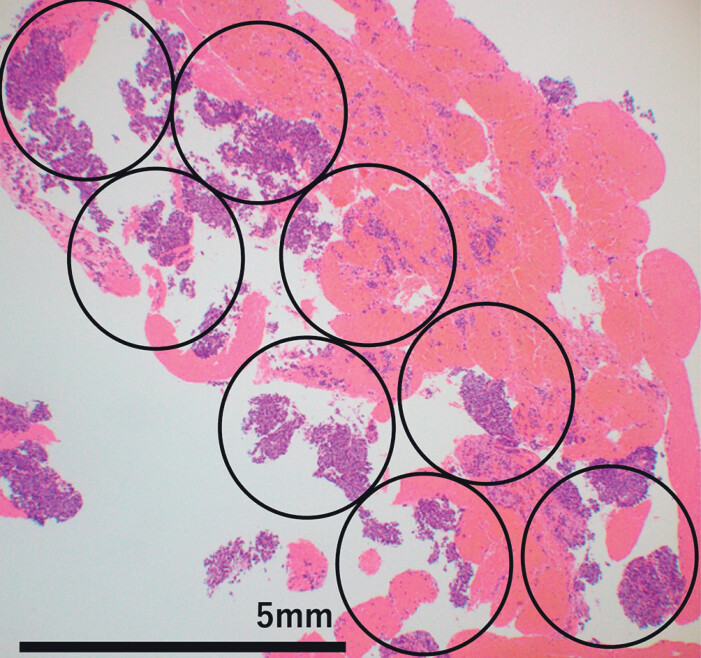
Example of an ideal specimen for comprehensive genome profiling (CGP). The section is stained with hematoxylin and eosin. The solid black circle represents one field of view at 10 × magnification (diameter, 2.2 mm). Because an ideal CGP specimen requires an area of at least 25 mm ^2^ containing tumor cells, we defined it as one that includes six or more 10 × fields containing tumor cell clusters. This specimen has six such fields, thereby meeting the criteria for an ideal specimen.

Pathological assessments, including cytological and histological evaluations, were performed by two experienced pathologists (YU and TO). Samples that were considered positive or suspicious for malignancy during either evaluation were classified as malignant. The final diagnosis was established based on surgical pathology results of the resected specimens or a positive malignancy diagnosis of the sample obtained using EUS-TA and confirmed by presence of a consistent clinical course for at least 6 months. To ensure suitability of the specimens for CGP, we evaluated the number of procured specimens using a previously reported scoring system 15 . According to the scoring system, an ideal sample was defined as one that comprised tumor cell clusters in six or more fields under ×10 magnification ( Fig. 1 ). Because the diameter of a field at ×10 magnification was 2.2 mm, a sample comprising tumor cell clusters in six or more such fields corresponded to an area that exceeded 25 mm ^2^ , which was the minimum tissue requirement for use with the F1CDx.

### Study outcomes

Patient data were collected from electronic medical records and endoscopy databases. The primary endpoint was the acquisition rate of ideal samples for CGP, and second endpoints were histological, cytological accuracy, and adverse events (AEs). In addition, we investigated factors that contribute to acquisition of ideal samples for CGP. AEs were defined and graded according to the American Society for Gastrointestinal Endoscopy Severity Grading System 16 .

### Statistical analysis

Continuous variables (expressed as medians and ranges) were compared using the Mann-Whitney U test. Categorical variables (expressed as proportions) were compared using Fisher’s exact test. Associations between adequacy of ideal samples for CGP and patient-related characteristics, such as disease type (primary or metastatic), tumor size, number of punctures, and needle size, were evaluated by performing univariate analysis. The cutoff value for tumor size was determined based on median value. Subgroup analyses were performed to evaluate adequacy of ideal samples for CGP according to needle size. *P* < 0.05 was considered statistically significant. All analyses were conducted using R software version 3.4.1 (R Foundation for Statistical Computing, Vienna, Austria).

## Results

### Patient characteristics

Thirty-six patients were included in this study. Of them, 22 (61.1%) were male and 14 (38.9%) were female, and their median age was 69.5 years (range, 30–90 years) ( Table 1 ). Liver lesions in 12 (33.3%) and 24 (66.7%) patients were categorized as primary and metastatic, respectively. Among the primary liver lesions, intrahepatic cholangiocarcinoma was the most common type (7 patients; 19.4%), followed by hepatocellular carcinoma (3 patients; 8.3%) and perihilar cholangiocarcinoma (2 patients; 5.6%). Metastatic liver lesions were most frequently associated with breast cancer (7 patients; 19.4%), intrahepatic cholangiocarcinoma (5 patients; 13.9%), and pancreatic cancer (5 patients; 13.9%). Other primary cancers included colon (2 patients; 5.6%), lung (2 patients; 5.6%), and gallbladder cancer (1 patient; 2.8%), mucinous cystic neoplasm (1 patient; 2.8%), and gastrointestinal stromal tumor (1 patient; 2.8%).

**Table:** **Table 1** Patient characteristics.

	n = 36
Age, median, years (range)	69.5 (30–90)
Sex, male, n (%)	22 (61.1)
Disease, n (%)	
Primary liver lesions	
Intrahepatic cholangiocarcinoma	7 (19.4)
Perihilar cholangiocarcinoma	2 (5.6)
Hepatocellular carcinoma	3 (8.3)
Metastatic liver lesions	
Intrahepatic cholangiocarcinoma	5 (13.9)
Pancreatic cancer	5 (13.9)
Gallbladder cancer	1 (2.8)
Mucinous cystic neoplasm	1 (2.8)
Colon cancer	2 (5.6)
Breast cancer	7 (19.4)
Lung cancer	2 (5.6)
Gastrointestinal stromal tumor	1 (2.8)
Baseline demographic and clinical features are shown, including age, sex, and primary or metastatic liver tumor types. Data are presented as number (percentage); age is shown as median (range).	

### EUS-TA procedure

The EUS-TA procedure is presented in Table 2 . Target liver lesions were predominantly located in the left lobe and affected 30 patients (83.3%). Lesions were also found in the right lobe (3 patients; 8.3%) and caudate lobe (3 patients; 8.3%). Median lesion size was 31 mm (range, 11–79 mm). The primary puncture route was transgastric for 30 patients (83.3%), whereas the transduodenal route was used for six patients (16.7%). A 22G needle was most commonly used (27 patients; 75%). A 25G needle was used for six patients (16.7%). A 19G needle was used for three patients (8.3%). The number of punctures varied among patients. Two punctures were performed for the majority of patients (22 patients; 61.1%). However, three punctures were performed for 11 patients (30.6%) and a single puncture was suitable for three patients (8.3%).

**Table:** **Table 2** EUS-TA details.

	n = 36
Target liver lesion, n (%)	
Left lobe	30 (83.3)
Right lobe	3 (8.3)
Caudate lobe	3 (8.3)
Lesion size, median (range), mm	31 (11–79)
Puncture route, n (%)	
Transgastric	30 (83.3)
Transduodenal	6 (16.7)
Needle gauge	
19	3 (8.3)
22	27 (75)
25	6 (16.7)
Punctures, n (%)	
1	3 (8.3)
2	22 (61.1)
3	11 (30.6)
EUS-TA, endoscopic ultrasound-guided tissue acquisition.	

### EUS-TA outcomes

Diagnostic accuracy and outcomes of EUS-TA for all 36 patients were evaluated ( Table 3 ). Cytological analysis resulted in a diagnostic accuracy rate of 72.2% (26/36), whereas histological analysis resulted in a higher accuracy rate of 94.4% (34/36). Moreover, ideal samples for CGP were obtained from 23 patients (63.9%). AEs were not observed during or after the procedure; specifically, no instances of bleeding or perforation occurred.

**Table:** **Table 3** Outcomes of EUS-TA.

	n = 36
Diagnostic accuracy, n (%)	
Cytology	26 (72.2)
Histology	34 (94.4)
Ideal CGP sample, n (%)	23 (63.9)
Adverse events, n (%)	
Bleeding	0
Perforation	0
Diagnostic accuracy (cytology and histology), adequacy for CGP, and adverse events are shown. Data are presented as number (percentage). CGP, comprehensive genomic profiling; EUS-TA, endoscopic ultrasound-guided tissue acquisition.	

### Ideal samples

Univariate analysis of factors associated with acquisition of ideal samples for CGP was performed ( Table 4 ). Adequacy rates were compared across various patient and procedure characteristics. Regarding tumor locations, ideal samples were obtained from 62.5% and 66.7% of patients with primary tumors and metastatic tumors, respectively; the difference between these groups was not significant ( *P* = 1.0). Similarly, tumor size did not significantly affect the adequacy rate; samples from tumors ≤ 30 mm and > 30 mm achieved adequacy rates of 55.6% and 72.2%, respectively ( *P* = 0.49). Number of punctures performed did not significantly affect the adequacy rate. Adequacy rates of 66.7%, 63.6%, and 63.6% were observed with a single puncture, two punctures, and three punctures, respectively ( *P* = 1.0). In contrast, needle gauge used for EUS-TA was statistically significantly associated with adequacy rate ( *P* = 0.015). The highest adequacy rate was achieved with the 19G needle (100%). The 22G and 25G needles were associated with adequacy rates of 70.4% and 16.7% (significantly lower), respectively.

**Table:** **Table 4** Univariate analysis of success of acquiring ideal samples for CGP.

Factors	Adequate % (n/N)	*P*
Tumor location		1.0
Primary	62.5 (15/24)	
Metastasis	66.7 (8/12)	
Size of the tumor (mm)		0.49
≤ 30 mm	55.6 (10/18)	
> 30 mm	72.2 (13/18)	
Punctures		1.0
1	66.7 (2/3)	
2	63.6 (14/22)	
3	63.6 (7/11)	
Needle (gauge)		0.015
19	100 (3/3)	
22	70.4 (19/27)	
25	16.7 (1/6)	
Tumor location, tumor size, number of punctures, and needle gauge were analyzed. CGP, comprehensive genome profiling.		

### Subgroup analysis

**Fig. 2:**
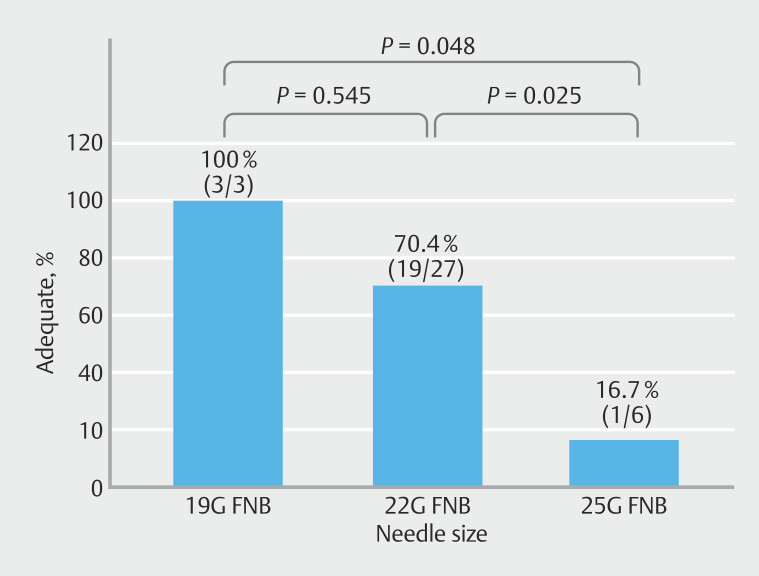
Success rates of acquiring ideal samples with different needle gauges for comprehensive genome profiling (CGP). Success rates of obtaining CGP-suitable samples were 100% with the 19G needle, 70.4% with the 22G needle, and 16.7% with the 25G needle. The 19G and 22G needles achieved success rates that were significantly higher than that of the 25G needle ( *P* = 0.048 and *P* = 0.025, respectively).

Fig. 2 illustrates the relationship between needle gauge and rate of acquiring ideal samples for CGP. An analysis revealed that the 19G needle achieved the highest success rate, with 100% of samples (3/3) classified as ideal for CGP. The rate of acquiring ideal samples for CGP with the 19G needle was not significantly different from that for the 22G needle (70.4%; 19/27; *P* = 0.545); however, it was significantly higher than that for the 25G needle (16.7%; 1/6; *P* = 0.048). In addition, the 22G needle demonstrated a significantly higher success rate than that for the 25G needle ( *P* = 0.025).

## Discussion

This retrospective study evaluated utility of EUS-TA for obtaining ideal liver tumor samples for CGP. Although EUS-TA is widely used for diagnostic purposes, its role in CGP remains unexplored. To the best of our knowledge, only a few studies have investigated the relationship between EUS-TA and CGP for liver tumor biopsy.

In this study, EUS-TA using FNB needles for malignant liver tumors demonstrated a histological diagnostic accuracy of 100%. In addition, the ideal sample acquisition rate for CGP was 63.9%. These findings underscore that EUS-TA provides both accurate histological diagnoses and high-quality samples for genomic analyses; therefore, it is a valuable tool for personalized oncology.

Univariate analysis revealed that needle gauge significantly influenced the rate of acquiring ideal samples for CGP. Specifically, the 25G needle was associated with a significantly lower success rate, whereas the 22G needle achieved a combined success rate of 73.3%. These findings suggest that selecting larger-gauge needles (22G or 19G) is critical to optimizing EUS-TA with CGP for liver lesions. Furthermore, needle gauge is important to optimization of outcomes. Needles with a gauge of 22 or larger are likely to enhance effectiveness of EUS-TA for genomic applications for liver lesions. Our findings underscore that, given the unique histological features of liver tumors—including frequent necrosis and cellular heterogeneity—liver-specific analyses are warranted and may provide procedure guidance distinct from that established for pancreatic tumors.

CGP has been recommended in National Comprehensive Cancer Network guidelines 17 ; however, significant challenges are associated with its implementation, particularly the limited number of eligible samples and variability in the pre-evaluation criteria among institutions. These inconsistencies can create disparities in CGP outcomes and restrict the number of cases eligible for evaluation. CGP involves substantial medical costs; therefore, it is crucial to establish stringent pre-evaluation conditions to ensure the adequacy of samples and minimize failures during actual CGP. Variability in pre-evaluation standards across institutions exacerbates this issue, resulting in inconsistent CGP results and limiting the pool of cases that can be effectively analyzed. Standardizing pre-evaluation criteria across institutions may optimize the CGP success rate and broaden applicability of CGP in clinical practice. Previous studies of CGP for pancreatic ductal adenocarcinoma have reported variable success rates (39.2%-97.0%) 5 6 7 8 9 . This variability underscores the importance of accurately predicting the likelihood of CGP success based on tissue samples obtained before the pre-evaluation. Because of the expense and limited availability of CGP, establishing reliable predictors of CGP success using initial tissue specimens is essential to optimizing resource allocation and reducing risk of failed analyses. To address these challenges, Kaneko et al. developed a pathological scoring system that calculates the required tissue area based on the criteria for use with the F1CDx to predict success of CGP 15 . In addition, Ishikawa et al. validated the utility of this scoring system and reported that use of ideal samples defined by this system significantly improved the CGP success rate using the F1CDx (94.1% vs. 55.0%) 18 . Standardized evaluation methods are vital to ensuring CGP success and optimizing resource utilization in clinical practice.

Limited reports of EUS-TA with CGP for liver tumors are available. A study that focused on biliary tract cancer reported a CGP-suitable specimen acquisition rate of 84.4% (27/32) for liver tumors using EUS-TA; however, detailed analyses were not performed 19 . In the present study, the rate of ideal sample acquisition for CGP was slightly lower (63.9%). A potential reason for this difference could be the needle size used during EUS-TA, which may have influenced adequacy of the collected specimens. By performing multivariate analysis, Ikeda et al. found that use of 19G needles and EUS-FNB were significant factors associated with obtaining specimens ideal for CGP 10 . Similarly, a retrospective study by Park et al. examined 190 patients who underwent EUS-TA for pancreatic tumors and reported that the success rate for CGP with a 25G needle was markedly lower than that with a 19G or 22G needle (38.8% vs. 60.9%; *P* = 0.003) 5 . The current study also demonstrated a significant association between needle gauge and acquisition of CGP-suitable specimens. Notably, the success rate with a 25G needle was significantly lower than that with a 19G or 22G needle. This observation was consistent for both pancreatic and liver tumors, suggesting that larger-gauge needles more effectively secure adequate specimens for CGP. In contrast, use of a 22G FNB needle achieved a CGP-suitable specimen acquisition rate of 70.4% (19/27) during this study. A previous investigation using the same scoring system reported a lower acquisition rate of 52.4% for pancreatic tumors sampled using a 22G FNB needle 18 . These findings suggest that 22G FNB needles may be more effective for liver tumors than for pancreatic tumors when obtaining CGP-suitable specimens. One possible explanation for this difference is the fibrotic nature of pancreatic cancer, which is associated with the challenging collection of ideal tissue samples. In contrast, liver tumors may allow collection of larger tissue volumes, thereby increasing the likelihood of obtaining samples ideal for CGP.

Nguyen et al. first reported use of EUS-TA for focal liver tumors in 1999 20 . Since then, a high diagnostic accuracy rate (range 89.7%-100%) and low AE rate (0%-6%) have been reported for this approach 20 21 22 23 24 25 26 27 . Consistent with these findings, the present study demonstrated a histological diagnostic accuracy rate of 100% with no reported AEs, thus reaffirming the reliability and safety of EUS-TA for liver tumors. EUS-TA is a valuable diagnostic modality for liver tumors. Recently, EUS has gained increasing attention in the field of hepatology; therefore, incorporating EUS-TA with CGP of liver tumors could have significant clinical implications. Because application of EUS-TA with CGP for liver tumors is an important topic, further investigations are required.

This study had several limitations. First, its retrospective design inherently introduced potential bias, which may have affected generalizability of the findings. Second, the small sample size limited the statistical power and robustness of the conclusions. Moreover, our cohort predominantly comprised left-lobe tumors (83.3%) and caudate-lobe tumors (8.3%). This distribution reflects our clinical practice, in which percutaneous biopsy is often selected for right-lobe lesions due to the limited reach and angulation constraints of EUS. Consequently, generalizability of our findings to right-lobe lesions is limited. Future prospective studies comparing modality selection and outcomes (diagnostic accuracy, CGP adequacy, and safety), including percutaneous biopsy, are warranted.

Third, success of CGP relies on the tumor tissue area and the tumor-to-nuclei ratio. During this study, the tumor-to-nuclei ratio was not specifically evaluated. However, because the tumor-to-nuclei ratio is strongly influenced by tumor characteristics, it may not be easily improved using sampling techniques. Therefore, our focus should be maximizing the tumor tissue area, which can be achieved through methodological optimization. Fourth, the study outcomes were not based on the final success rates of CGP; rather, they were evaluated using surrogate markers. Therefore, the final CGP success rates may differ from previously reported results. However, the validity of this evaluation method has been demonstrated in previous studies 18 , and findings from this study are consistent with practical considerations in routine clinical practice.

## Conclusions

In conclusion, EUS-FNB for liver tumors demonstrates potential utility not only for histological diagnosis but also for CGP. In addition, use of 22G or larger needles may enhance the success of obtaining adequate samples for CGP.
